# Theta, Mental Flexibility, and Post-Traumatic Stress Disorder: Connecting in the Parietal Cortex

**DOI:** 10.1371/journal.pone.0123541

**Published:** 2015-04-24

**Authors:** Benjamin T. Dunkley, Paul A. Sedge, Sam M. Doesburg, Richard J. Grodecki, Rakesh Jetly, Pang N. Shek, Margot J. Taylor, Elizabeth W. Pang

**Affiliations:** 1 Department of Diagnostic Imaging, Hospital for Sick Children, Toronto, Canada; 2 Neuroscience & Mental Health Program, Hospital for Sick Children Research Institute, Toronto, Canada; 3 Directorate of Mental Health, Canadian Forces Health Services, Ottawa, Canada; 4 Department of Medical Imaging, University of Toronto, Toronto, Canada; 5 Department of Psychology, University of Toronto, Toronto, Canada; 6 Canadian Forces Environmental Medicine Establishment, Toronto, Canada; 7 Defence Research and Development Canada, Toronto, Canada; 8 Division of Neurology, Hospital for Sick Children, Toronto, Canada; Osaka University Graduate School of Medicine, JAPAN

## Abstract

Post-traumatic stress disorder (PTSD) is a mental health injury characterised by re-experiencing, avoidance, numbing and hyperarousal. Whilst the aetiology of the disorder is relatively well understood, there is debate about the prevalence of cognitive sequelae that manifest in PTSD. In particular, there are conflicting reports about deficits in executive function and mental flexibility. Even less is known about the neural changes that underlie such deficits. Here, we used magnetoencephalography to study differences in functional connectivity during a mental flexibility task in combat-related PTSD (all males, mean age = 37.4, n = 18) versus a military control (all males, mean age = 33.05, n = 19) group. We observed large-scale increases in theta connectivity in the PTSD group compared to controls. The PTSD group performance was compromised in the more attentionally-demanding task and this was characterised by 'late-stage' theta hyperconnectivity, concentrated in network connections involving right parietal cortex. Furthermore, we observed significant correlations with the connectivity strength in this region with a number of cognitive-behavioural outcomes, including measures of attention, depression and anxiety. These findings suggest atypical coordination of neural synchronisation in large scale networks contributes to deficits in mental flexibility for PTSD populations in timed, attentionally-demanding tasks, and this propensity toward network hyperconnectivity may play a more general role in the cognitive sequelae evident in this disorder.

## Introduction

Post-traumatic stress disorder (PTSD) is a serious mental health injury described by anxious and depressive features which develop after exposure to a traumatic life event(s). PTSD is defined in the Diagnostic and Statistical Manual of Mental Disorders (DSM-V) as being comprised of a four symptom cluster of re-experiencing, avoidance, emotional numbing and hyper-vigilance/arousal [1]. Approximately 50% of the general population experience at least one traumatic event during their lifetime, but the incidence of the disorder as a whole is around 5–10% [2]. However, in military populations the prevalence of PTSD is higher, especially in those returning from recent combat deployments in the Afghanistan [3,4].

Cognitive sequelae are often evident in PTSD populations. Although there is ongoing debate regarding pervasive changes in cognitive functioning, many studies report functional deficits in a number of domains, including short-term working memory [5,6,7], sustained attention [8,9,10], inhibition [9,10,11], memory recall [6,12], executive function [9,13,14] and emotional processing [14,15]. Despite this, little is known about the neurobiological bases of such deficits.

There are conflicting reports of deficits in mental flexibility in PTSD, with some researchers reporting difficulties in cognitive control for attentional, fast-paced task-switching in the Trail Making Task[9,16], whilst others do not [11,17,18]. It is also suggested that, for the most part, rule-based, untimed tasks that require planning and strategy-switching are largely unaffected (for a review see [19]). However, given the heterogeneity of PTSD populations and its triggers, pre-existing cognitive risk-factors, its comorbidity with a number of cognitive sequelae, and the multitude of methods for assessing mental flexibility, formulating a unified hypothesis about the impact of the disorder on executive function has remained elusive. This is an area that requires more research; with the adaptation and integration of well-established behavioural paradigms with advanced imaging approaches, we can probe the neural structures and functional changes that are associated with such cognitive deficits seen in PTSD.

The structures implicated in cognitive control and attentional set-shifting include principally the prefrontal cortex (PFC), the anterior cingulate cortex (ACC) and the posterior parietal cortex (PPC) [20–23]. However, less is known about the functional connectivity required for these tasks and how the ongoing spatiotemporal integration of information among these regions contributes to observed behavioural differences in performance.

At the network level, cognitive control likely requires efficient routing of task-relevant information and the inhibition of task-irrelevant regions, and these mechanisms may be critical in effective mental flexibility needed for complex goal-directed behaviour. One proposed mechanism for such functional interactions among brain areas is the synchronisation of neural oscillations between disparate brain regions, which is thought to allow the effective routing and temporal coordination of information required for cognitive flexibility [24,25,26] The neuroimaging technique optimal for noninvasively assessing spatio-temporal patterns in brain connectivity is magnetoencephalography (MEG), as it has the millisecond timing resolution of electroencephalography (EEG), but a far better spatial resolution than EEG, in the range of 5 mm, which is approaching that of functional magnetic resonance imaging (fMRI).

Neural synchrony has been widely implicated in cognition, perception and action, and inter-regional synchronisation is thought to be a mechanism that mediates functional connectivity of within-brain networks [24,27]. Additionally, altered patterns of inter-regional synchrony have been observed in numerous neuropsychiatric populations, and mapping these atypical networks has proved fruitful in understanding cortical pathophysiology, with aberrant connectivity thought to play a significant role in a number of disorders [28,29]. In resting state studies, enhanced MEG slow-waves in left temporal and right frontal regions, and decreased oscillations in right parietal cortex [30] are prominent in PTSD. Georgopoulos et al. [31] demonstrated that atypical patterns of abnormal synchronous oscillations are able to differentiate PTSD from control subjects, and that ineffective communication between right temporo-parietal areas and other brain regions might underlie aspects of the disorder [32]. This group also reported negative correlations between neural oscillations in the right superior temporal gyrus, with resilience to lifetime trauma in control veterans, but not those with PTSD [33], and recently, we have shown that high-frequency gamma synchronisation (80–150 Hz) differentiates PTSD from control soldiers, with network strength measures in the left hippocampus correlating with symptom severity [34]. These studies collectively suggest abnormal coherency in brain oscillations might contribute to some of the symptoms and cognitive sequelae of the disorder. However, knowledge remains scant regarding task-dependent changes in connectivity in PTSD populations.

Here, we examined cognitive flexibility in a fast-paced, attentionally-demanding, set-shifting task in a PTSD population and matched control group whilst undergoing MEG recording. We explored task-dependent, frequency-specific interactions in source-resolved networks using a source-resolved inter-regional phase synchronisation approach, network statistics and graph theoretical analysis. We hypothesised that the PTSD participants would display atypical network connectivity related to the task, and that topological measures from critical regions would correlate with individual differences in performance. Relations between atypical task dependent network synchronisation and cognitive sequelae were investigated in the PTSD group.

## Results

### Cognitive-behavioural assessment

Measures on the cognitive-behavioural tests and clinical assessments, as well as the characteristics of the soldiers with and without PTSD, are presented in Table 1.

**Table 1:** Cognitive-behavioural test measures for PTSD and Control groups, with SD in parentheses, and inferential tests, with *df* in parentheses.

	PTSD	Control	Test statistic
*n*	18	19	
Age	37.38 (6.69)	32.21 (4.60)	*t*(35) = -2.75, *p* = 0.009
WASI	108.67 (14.07)	115.63 (13.72)	*t*(35) = 1.52, *p* = 0.14
AUDIT	8.06 (6.68)	6.05 (3.69)	*t*(35) = -1.14, *p* = 0.26
Conners	23.72 (9.06)	7.26 (5.18)	*t*(35) = -6.83, *p*<0.001
GAD-7	15.00 (4.26)	1.73 (1.94)	*t*(35) = -12.31, *p*<0.001
PHQ9	17.17 (4.11)	1.74 (2.40)	*t*(35) = -14.05, *p*<0.001
PCL	62.188 (7.24)	N/A	

WASI, *Wechsler Abbreviated Scale of Intelligence*; AUDIT, *Alcohol Use Disorders Identification Test;* Conners, *Attention-Deficit Hyperactivity Disorder Test;* GAD-7, *Generalized Anxiety Disorder 7;* PHQ9, *Patient Health Questionnaire;* PCL, *Post Traumatic Stress Disorder Check List*.

### Behavioural results

**Fig 1:**
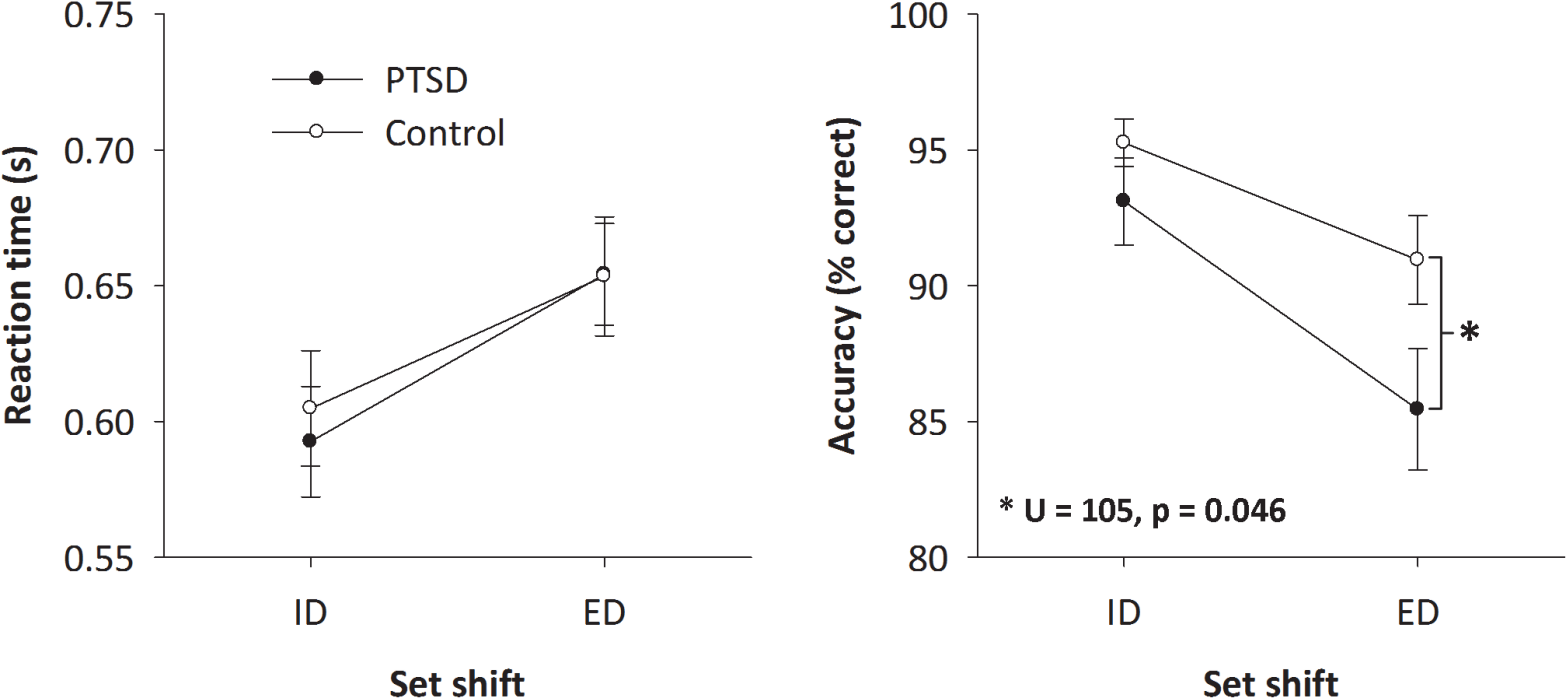
Mean behavioural results (reaction time and accuracy) in the Intra and Extra-Dimensional set-shifting tasks for PTSD and control soldiers, with ±1 SE bars. There were no differences between-groups for reaction time, although RT was longer for the Extra compared to Intra-dimensional shifts. No significant differences were observed between the groups for accuracy in the Intra-dimensional shifts; however, the PTSD group had significantly poorer performance in the Extra-dimensional set-shifting task (*p<0.05).

Behavioural results for the tasks are shown in Fig 1. Measures of reaction time and task accuracy were separately submitted to a 2x2 mixed factorial ANOVA with ‘Group’ (PTSD and control) as the between-participant variable and ‘Condition’ (Intra- and Extra-dimensional) as the within-participant variable. Extra-dimensional shifts required longer reaction times (654 ± 87 ms) than Intra-dimensional shifts (599 ± 89 ms) (*F*(1,35) = 69.69, *p*<0.001). There was no significant difference between groups in reaction time or any significant interaction effect.

Task accuracy was higher in the Intra-dimensional shifts (94.22% ± 0.90) than in the Extra-dimensional shifts (88.27% ± 1.43) (*F*(1,35) = 24.24, *p*<0.001), and the PTSD group (89.28% ± 1.50) tended to perform worse than the control soldiers (93.11% ± 0.98) (*F*(1,35) = 3.75, *p* = 0.06). Tests of normality revealed accuracy distribution was significantly non-normal (non-Gaussian) for all groups and shifts when tested separately (Shapiro-Wilk; *p*<0.05), displaying a high-degree of negative skewness. Given this, post-hoc testing was conducted using non-parametric statistics (Mann-Whitney/Wilcoxon Rank Sum), which revealed that there was a significant group difference only in the Extra-dimensional task (Mann-Whitney, *U* = 105, *p* = 0.046).

**Fig 2:**
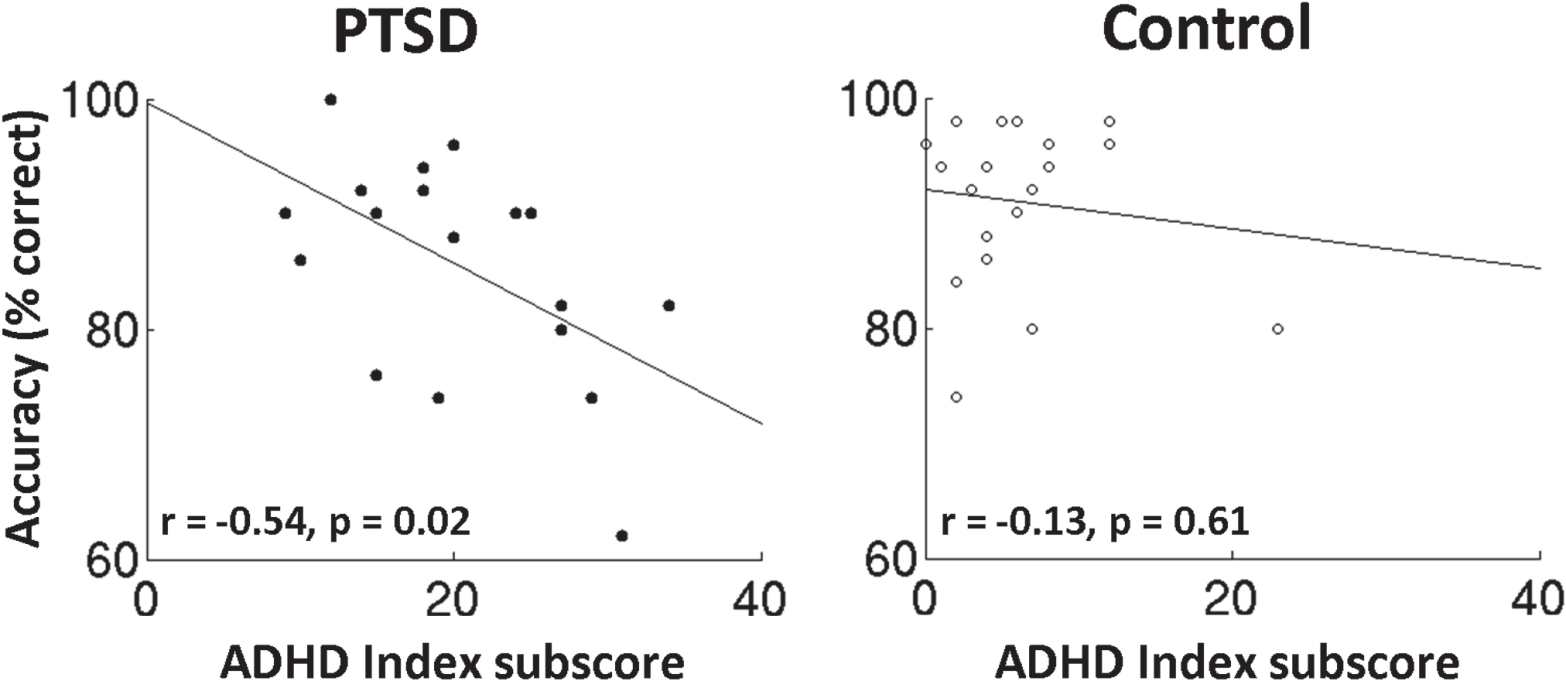
ADHD Index subscores on the Conners test correlates with Extra-dimensional set-shifting task accuracy for the PTSD group (*p*<0.05), but not for the Control group (*p*>0.05).

Additionally, the ADHD Index subscore of the Conners test and Extra-dimensional accuracy were also found to negatively correlate in the PTSD group (*r* = -0.54, *p* = 0.02; Fig 2, *left panel*)—accuracy decreased as ADHD Index increased—but not in the Control group (*r* = -0.13, *p* = 0.61; Fig 2, *right panel*). This relation was not observed in any of the other behavioural assessments.

### Task-dependent connectivity analyses

**Fig 3:**
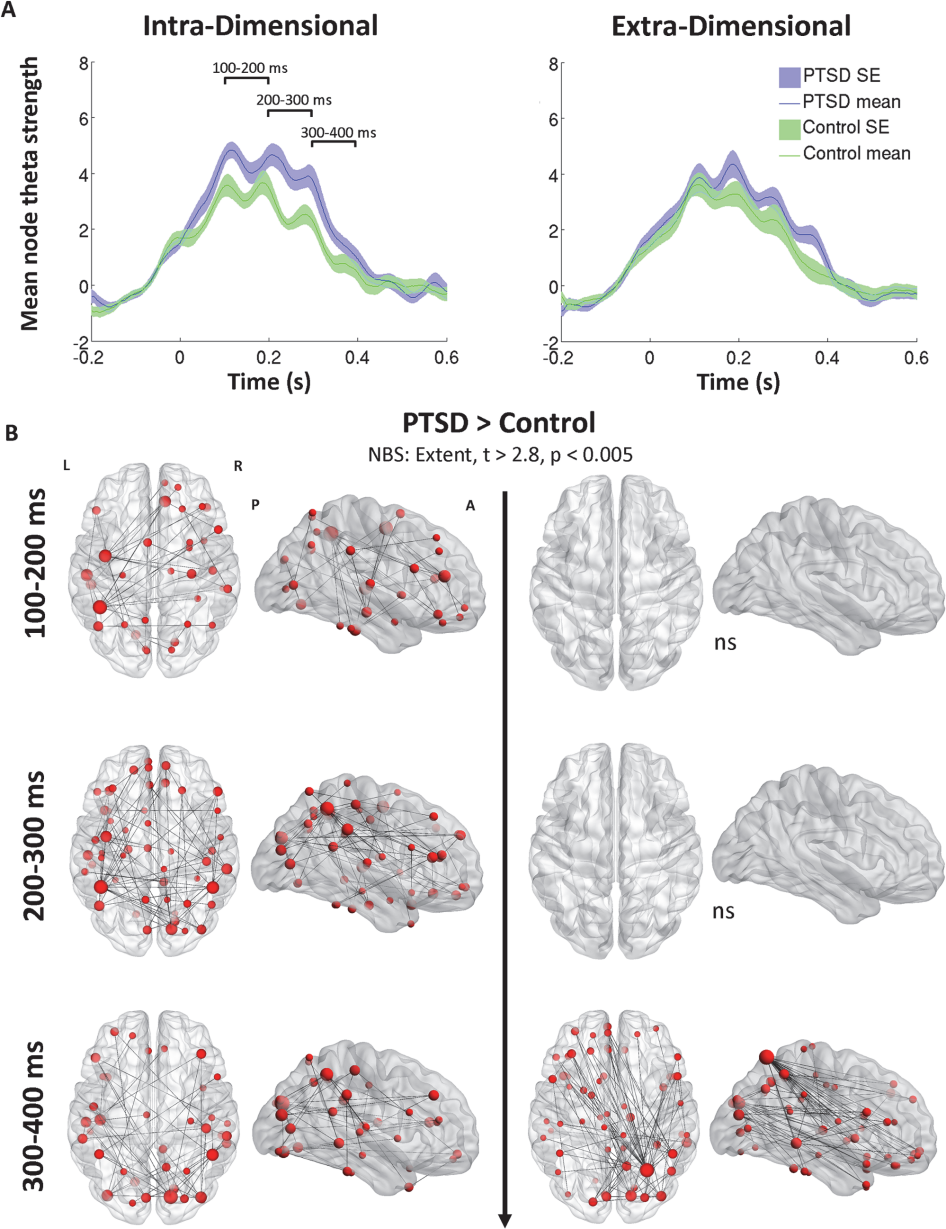
Whole-brain theta connectivity time-series and the temporal evolution of functional network differences between PTSD and control groups. **(A)** The PTSD group show elevated whole-brain theta coherence during the Intra-dimensional task (*top left panel*), particularly in the 100–300ms time window, which is evident in the regional connectivity maps below (**B;** all maps PTSD > control). In the Extra-dimensional set shifting task, the groups do not differ until the 300–400 ms time-window, when the PTSD show high-degree node differences, particularly in the right superior parietal lobule connecting to the right temporal and left frontal regions.

Mean node connectivity strength increases in theta (4–7 Hz) task-dependent network coherence were observed in both the PTSD (blue traces) and control soldiers (green traces) in both the Intra-Dimensional (*top left panel*) and Extra-Dimensional (*top right panel*) set-shifting tasks (Fig 3A).

For the Intra-dimensional set-shift time-series, there was a clear increase in task-dependent connectivity for the PTSD group compared to controls in the 100–300 ms time window after stimulus onset, after which both groups slowly returned to baseline levels by 400ms post-stimulus. For the Extra-dimensional set-shift, differences between the groups in connectivity were more subtle, with a higher mean node strength in the PTSD group at the 300–400ms time-window, consistent with a slower return-to-baseline connectivity strength, occurring by the 500ms time-point. Task-dependent changes in connectivity were also observed in the alpha (8–14 Hz) and beta (15–30 Hz) frequency ranges, but as no global differences were noted between the groups, they were not investigated further.

To explore the temporal evolution of connectivity changes between the groups, averaged adjacency matrices were computed for non-overlapping 100 ms time-windows (100–200, 200–300, 300–400 ms), selected on the basis of visual inspection of network connectivity (see Fig 3A), and baselined against the pre-stimulus 200ms time-window for each participant, partitioned into groups, and evaluated using the Network Based Statistic thresholded at a *t*-statistic ≥2.8 (*p* = 0.004). Initial univariate thresholds were adapted to data distributions being compared, as recommended by Zalesky et al. [43]. As this threshold is applied to both the real and surrogate data, correction for false positives due to multiple comparisons was achieved across thresholds.

Functional connectivity maps in axial and sagittal orientations can be seen in Fig 3B, with the size of the node scaled to the magnitude of the significant connectivity difference between the groups; significant edges are also marked as interconnecting lines between AAL seed regions. For Intra-dimensional shifts in the 100–200 ms time window, results revealed a distributed network of brain regions that were hyperconnected in the PTSD group, with differences observed in the left parietal, left temporal and left precentral cortex, as well as right medial frontal regions. For the 200–300 ms time window, these differences encompassed regions in the right occipital cortex, left and right parietal cortices, left temporal and left precentral regions. For the final 300–400 ms time window, high-strength node differences were noted in right occipital cortex, right parietal and left temporal regions.

Consistent with the time-series for the Extra-dimensional set-shifting, NBS connectivity analyses confirmed there were no significant differences between the groups in the 100–200 ms and 200–300 ms periods. Despite the conservative initial thresholding (*t*≥2.8, *p* = 0.004), there were still no differences found when the threshold was relaxed to *t* = 1.69, *p* = 0.05. However, for the 300–400 ms time window, results revealed a hyperconnected node in the PTSD group located in the right superior parietal lobule (SPL). This node was over-connected to approximately 30% of the seeds in the network (with a degree difference of 26) compared to the control group, and was connected to a distributed network of seeds across the brain, particularly in the left frontal and right middle temporal regions. Given this region’s importance in the connectivity differences between the groups, time-series of graph properties describing the task-dependent network engagement of this region were examined in more detail.

### Right parietal hyperconnectivity, task performance and cognitive-behavioural outcomes

**Fig 4:**
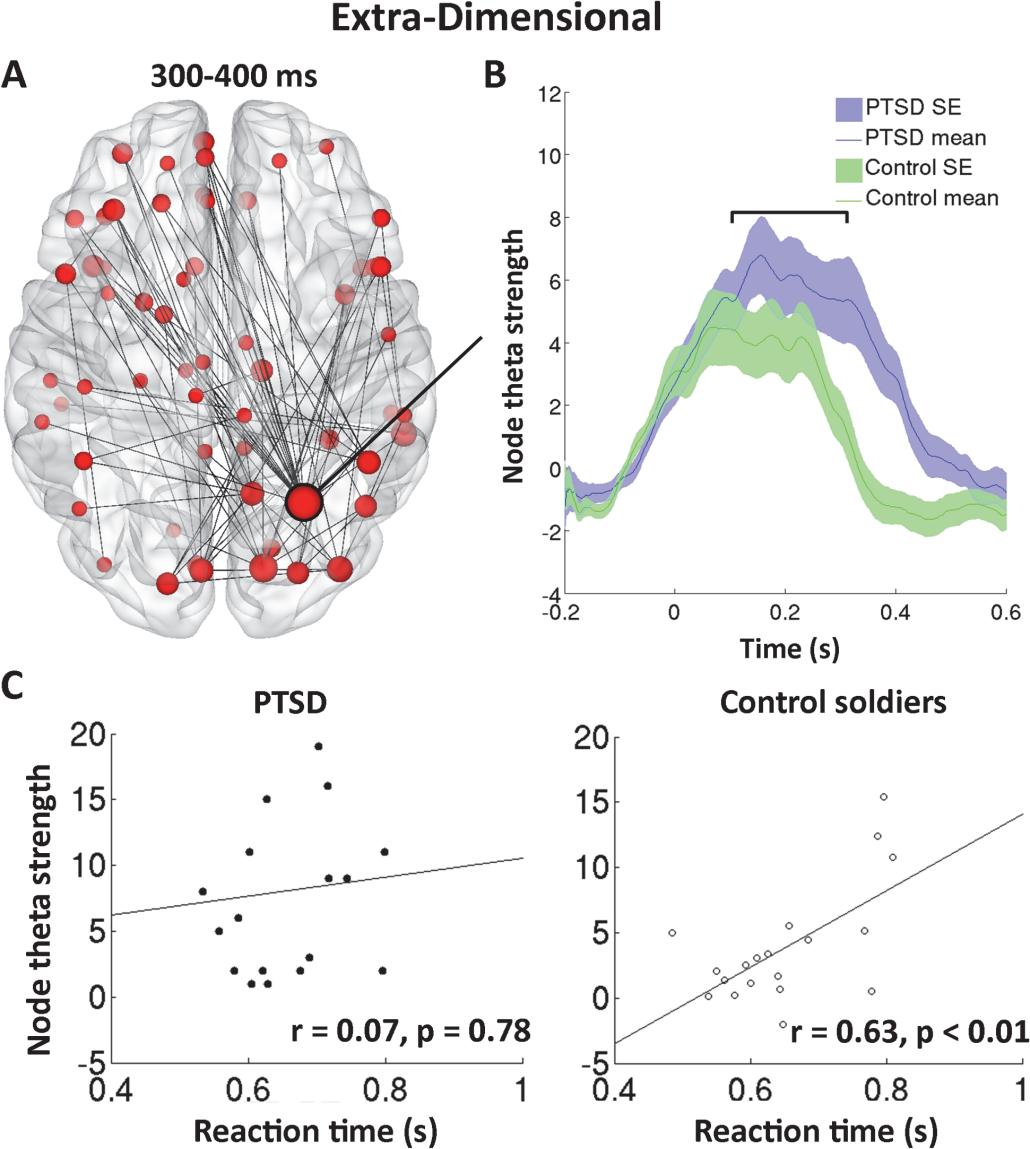
Atypical network theta connectivity in the right superior parietal cortex seed (A, B), and reaction time correlations in the Extra-Dimensional task (C). The PTSD group show no significant correlation of theta strength with reaction time (*bottom left panel*) in the 100–300 ms time-window, whilst there is a strong significant correlation in the control group (*bottom right panel*). Connectivity time-series were baselined against the -200–0 ms time window and smoothed using a 50 ms moving-average for visualisation purposes; all further analyses were conducted on unsmoothed connectivity time-series.

Fig 4A shows functional connectivity differences in the theta band for the Extra-dimensional shifts in the 300–400 ms time window (*top left panel*) and extracted connectivity strength time-series from the right superior parietal node (100 ms moving-average time-window for visualisation; analyses are on unfiltered data, Fig 4B). Both groups showed steep initial increases in theta connectivity strength following stimulus presentation, with the control group peaking at ~50 ms, whilst the PTSD group continued to increase until around ~150 ms. At this stage theta coherence plateaus for both groups and remains largely stable for the next 200 ms. Following this, the control group connectivity swiftly declines at around 250 ms to approximately baseline levels at 350 ms. The PTSD group however showed a delayed latency in this ‘return-to-baseline’, with decreases not observed until ~350 ms and reaching baseline at ~450 ms.

To determine whether connectivity in this region played a task-specific role in the observed individual differences in task performance, correlations of node strength against reaction time and accuracy in the Extra-dimensional task were computed. Given that we were interested in examining individual variability, rather than between-group differences in performance, we chose a time-window of 100–300 ms (shown in Fig 4B) from which to extract mean connectivity, which encompasses the plateau shown in both groups for connectivity coherence. Connectivity strength in this right parietal node did not correlate with accuracy (all *p*>0.05) in either group, but did correlate highly with Extra-dimensional reaction time in the control group (*r* = 0.63, *p* = 0.0042), but not in the PTSD group (*r* = 0.07, *p* = 0.78) (Fig 4C).

**Fig 5:**
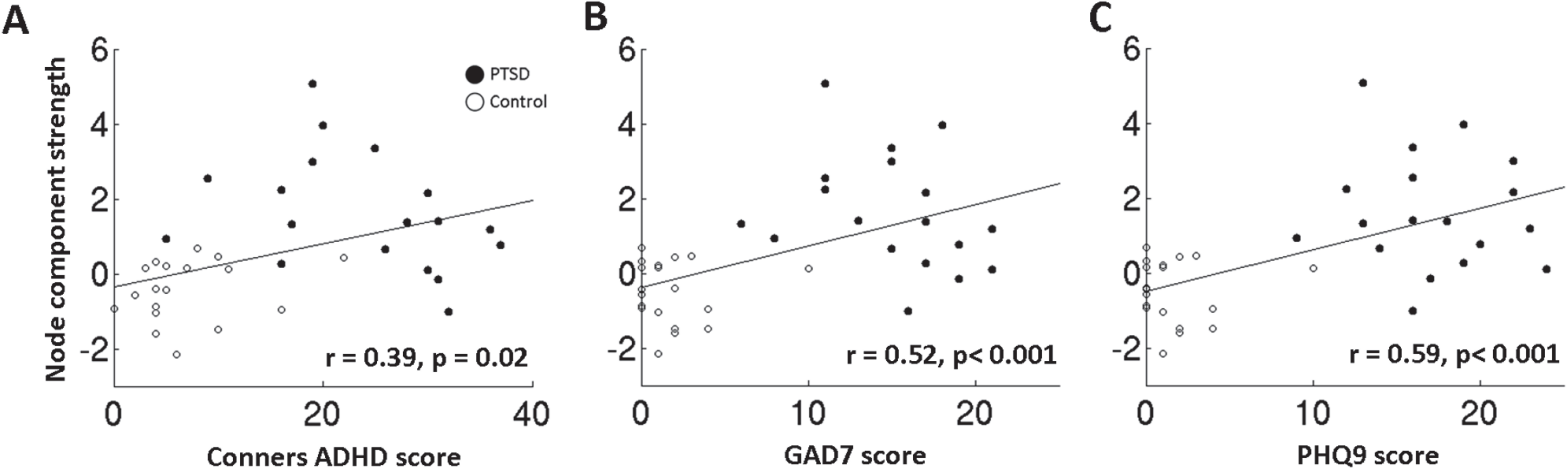
Right parietal theta in the Extra-dimensional task correlates with combined group cognitive assessment in the Conners ADHD, GAD7 and PHQ9 test scores. **(A)** Scatterplot showing a significant positive correlation between right superior parietal lobule theta connectivity strength *within* the hyperconnected network component during the more difficult Extra-dimensional set-shifting task in the 100–300 ms time window versus Conners ADHD test score, at the combined group level. **(B)** Same as before, but for the GAD7, **(C)** and for the PHQ9.

Furthermore, significant correlations of theta strength *within* the atypical network versus a number of cognitive-behavioural assessment outcomes were also found (Fig 5). Interestingly, moderate significant correlations were found for theta connectivity versus the Conners scale for attention deficits (*r* = 0.39, *p* = 0.02; Fig 5, *left panel*), GAD7 (*r* = 0.52, *p*<0.001, *middle panel*), and PHQ9 (*r* = 0.59, *p*<0.001, *right panel*).

## Discussion

This study provides evidence of atypical theta network synchronisation during a task requiring attentional control and cognitive flexibility in a PTSD population. Soldiers with combat-related PTSD displayed theta band hyperconnectivity during the set-shifting tasks compared to matched military controls who did not develop PTSD. The PTSD group exhibited distributed network-wide increases in theta synchrony during an Intra-dimensional set-shifting task and late-stage hyperconnectivity centred on the right parietal cortex connected to frontal and temporal regions during the more attentionally-demanding Extra-dimensional task.

Furthermore, we found that measures of network topology in the right superior parietal lobule, ‘the crossroads of the brain’ [47] and a region implicated in cognitive control and attention, was associated with individual variability in reaction time in the control but not in PTSD group. This indicates that recruitment of neurophysiological interactions in distributed brain networks in support of cognitive performance is atypical in soldiers with PTSD. Additionally, we found that parietal theta connectivity strength within the atypical network was positively correlated with an outcome measure of attentional deficits when the groups are combined, as well as measures of depression and anxiety. Together, these results suggest that right parietal cortex connectivity in the theta band plays a critical role in the temporal coordination of information required for effective cognitive flexibility, and that enhanced theta synchronisation plays a more general role in attentional, depressive and anxiety-related sequelae observed in PTSD populations.

### Set-shifting deficits in PTSD

In terms of behavioural performance, both groups showed similar reaction times, with increased response latencies in the Extra-dimensional compared to the Intra-dimensional condition, as expected (see [38]). Furthermore, measures of task accuracy were similar in the ID condition, with almost all participants operating close to ceiling. However, for the more difficult, attentionally-demanding ED task, there was a significant decrease in accuracy for the PTSD group compared to the controls. This poorer performance is in line with some of the literature which suggests that those with PTSD have deficits in cognitive control and mental flexibility for fast-paced task-switching [9,16,19].

### Theta connectivity involving right parietal cortex

Network connectivity group differences were observed during task processing (100–400 ms post-set-shift), with PTSD patients showing elevated large-scale theta synchronisation encompassing frontal, temporal and parietal regions. These differences in connectivity suggest that PTSD populations require heightened coordination of task-relevant brain regions to maintain the same degree of performance, or that hyperconnectivity is epiphenomenal and unrelated to goal-directed behaviour in this case. In other words, PTSD could be associated with some general, chronic, enhanced theta, due to ongoing, atypical network interactions, rather than task-specific processing. From this perspective neural synchrony served to selectively integrate relevant neural populations to support task performance, and accordingly soldiers with PTSD may not be able to recruit sufficiently *selective* inter-regional communication, resulting in less efficient networks supporting task performance. However, we found that early differences in connectivity were not due to performance differences in the ID task; the PTSD population performed comparably whilst employing different patterns of connectivity. This might be due to compensatory reorganisation of function, mediated by theta synchrony.

Interestingly, unlike divergences observed in the ID task, for the ED task differential connectivity was absent for much of the duration and was seen only in the later stages of task-processing (300–400 ms). Disparities in ED performance, but not in connectivity (in the 100–300 ms time window) suggest neural networks are more efficient at routing task-relevant information for goal-directed action in normal brain function. Analysis of the changes in connectivity in the later window (300–400 ms), however, revealed large-scale alterations in theta coherence, with the PTSD group showing significant network connectivity abnormalities in regions often implicated in the disorder, such as the frontal [48–51], temporal [30,32,33] and parietal regions [30], largely consistent with those generally reported in the literature [52]. Critically, these are areas shown to be active in normative studies on mental flexibility and cognitive control in set-shifting tasks [20,21,38,53,54,55].

We observed a prominently hyper-synchronised node in the right parietal region, specifically the superior parietal lobule (SPL), a region critically involved in the fronto-parietal attentional control network (for a review see [47]). Whilst classically thought of as involved in spatial attention [56,57], the SPL is also activated by attentional shifts between changes in the dimensionality of stimuli [57,58,59]. Furthermore, studies have shown impaired set-shifting performance following lesions to parietal cortex in animals [60], consistent with our finding that atypical connectivity patterns centred in right parietal regions may play a significant role in set-shifting deficits in PTSD.

Theta oscillations are thought to play a major mechanistic role in cognition, sensation and action, with studies relating them to central executive function [61,62], working memory [63,64,65], as well as sensorimotor integration [66] and task-switching [67]. Here, we observed that lower theta synchronisation facilitated reaction time to extra-dimensional rule changes in controls, but not in PTSD. This suggests that *increased* synchronisation in normal brain function increases response time. This differential relation between theta synchronisation and reaction time in PTSD and controls perhaps suggests that the PTSD group suffer from ongoing, atypical theta connectivity that does not correlate linearly with changes in response time (in other words, we might observe an inverted ‘U’ of connectivity versus efficiency, because of existing theta hyperconnectivity). Importantly however, we show a significant correlation between right parietal connectivity strength at the theta frequency within the aberrant functional network and a number of neuropsychological indices at the group level, including measures of anxiety (GAD7), depression (PHQ9) and attentional deficits (Conners ADHD).

These results suggest that the right superior parietal lobule, which had the highest connectivity difference between the groups within this atypical network, has a critical role in selecting and integrating neural populations relevant for efficient task performance. Consistent with this idea, the theta coherence in this region during task performance was associated with some of the wider cognitive sequelae evident in PTSD. This novel finding significantly extends previous research on ongoing local neural synchrony/cortical oscillations [30] by exploring the network interactions with behavioural and cognitive assessment. Our results also go beyond studies that have shown only sensor-space measures of neural interactions are linked to PTSD, and shows that source-resolved activity in the theta band in this important multi-modal hub, implicated in cognitive flexibility and attentional control, correlates significantly with neuropsychological findings. Future longitudinal studies will be valuable in determining the applicability of these measures in monitoring intervention efficacy and long-term outcomes.

### Limitations

Limitations of the study centre on potential confounding variables that might explain the alterations in connectivity observed here. These include the significant difference in the presence of medication in the PTSD patient cohort, and the psychiatric comorbities. Given the naturalistic aim of the study, all PTSD patients were on evidence-based psychotropic medication(s), and it was not within our ethics approval to ask them to go off medications for a study. The presence of comorbid psychiatric factors in the PTSD group could contribute to the alterations in connectivity observed here. Future studies, with a larger sample, could address these issues.

Another point to consider would be whether the control group had experienced a similar number of traumatic event types as the PTSD group. It could be that trauma exposure was higher in the PTSD group compared to the combat-exposed non-PTSD group. Previous research has shown a dose-response relation of traumatic load on PTSD risk [68,69]. As we did not collect PTSD Check List scores in the control group, we cannot comment on the presence of sub-threshold PTSD symptomology. However, as there were significant differences between the groups, we interpret our results as reflecting a true group effect related to PTSD. Future studies could examine connectivity in a non-exposed civilian population, as well as PCL scores in trauma-exposed control groups.

### Conclusions

This study provides the first evidence for large-scale alterations in neurophysiological connectivity in a PTSD population undergoing a task involving cognitive flexibility. We show the important relation of theta connectivity and response times when reacting to task-related changes in a set-shifting paradigm. Finally, we demonstrate that this atypical increase in inter-regional theta synchronisation is associated with cognitive and affective symptomology of PTSD, such as anxiety and depressive symptoms, and in particular sequelae such as mental inflexibility, attentional control deficits and chronic hyperarousal.

## Materials and Methods

### Participants

MEG data were recorded from 23 Canadian Armed Forces soldiers, diagnosed with PTSD (all male, mean age = 37.4, SD = 6.8). Twenty-one soldiers (all male, mean age = 33.05, SD = 5.26), also from the Canadian Armed Forces, and confirmed not to have PTSD were recruited as a control group. Both groups of soldiers participated in the Afghan mission and were exposed to combat in frontline deployment roles; thus both groups had similar levels of combat exposure and military experience. Potential participants were invited to contact a member of the research team and were screened for PTSD according to the inclusion/exclusion criteria described below. If soldiers met criteria, they were invited to the Hospital for Sick Children MEG Lab where all participants underwent cognitive testing and completed a number of tasks in the MEG scanner. However, due to excessive motion and/or artefacts in the MEG data, five participants were excluded from the PTSD group, leaving 18, and two were excluded from the control group, leaving 19 (37 in total). All participants had normal or corrected-to-normal visual acuity. All research was approved by Research Ethics Board at the Hospital for Sick Children. All participants provided written informed consent to participate in the study, and this consent procedure was approved by the Research Ethics Board at the Hospital for Sick Children.

Participants were included in the PTSD group if they met the following criteria: they had a diagnosis of combat-related PTSD from an operational trauma stress support centre (OTSSC); PTSD symptoms were present from 1 to 4 years prior to participation in the study; they were engaged in regular mental health follow-up; they had moderate or greater severity (>50) on the PTSD check list. The diagnosis was determined by a psychiatrist or psychologist specializing in trauma-related mental health injuries and conducted through a comprehensive, semi-structured interview based upon DSM-IV-TR diagnostic criteria [35], along with Canadian Armed Forces (CAF) standardized psychometric testing. Interview-based clinician diagnosis of mental disorders is considered superior to pen and paper or self-administered screening methods. All participants in the PTSD group were recruited from one of the CAF OTSSCs, which are centres of excellence for the diagnosis and treatment of trauma-related mental health injuries. There were usually more than one DSM-IV-TR ‘A1’ stressor-related criteria [35] identified as a traumatic event contributing to the development of PTSD (direct personal experience of an event that involves actual or threatened death or injury), with diagnosis related to operational exposure. Control soldiers were carefully matched on rank, education level, handedness and military experience when compared with the PTSD group. They were stationed in Ontario (either at bases in Ottawa, Petawawa or Toronto), and were recruited through promotional materials (posters, leaflets, etc.) and announcements by commanders.

Additional inclusion criteria that were applied to both groups included: no history of a diagnosed traumatic brain injury (TBI) as screened by a psychiatrist through a review of the electronic health record, telephone interview, and administration of the Defence and Veteran's Brain Injury Centre (DVBIC) screening tool; English-speaking and able to understand task instructions and give informed consent. Exclusion criteria included ferrous metal inside the body or implanted medical devices that might be MRI contraindications or interfere with MEG data acquisition; seizures or other neurological disorders; certain ongoing medications (anticonvulsants, and/or benzodiazepines, or other GABA antagonists) known to directly or significantly influence EEG findings. As all the PTSD participants were being treated for PTSD, they were on evidenced-based psychotropic medication(s), such as selective serotonin reuptake inhibitors (SSRIs), serotonin-norephedrine reuptake inhibitors (SNRIs), and Prazosin.

### Cognitive-behavioural evaluation

To characterize the groups, all subjects completed short clinical assessments (the Wechsler Abbreviated Scale of Intelligence (WASI), the Conners scale, 3^rd^ ed. to identify attention disorder, the Alcohol Use Disorders Identification Test (AUDIT), the Generalized Anxiety Disorder 7-item Scale (GAD7), Patient Health Questionnaire (PHQ9) and the Post Traumatic Stress Disorder Check List (PCL)). These assessments were selected in consultation with our team psychiatrist as they capture domains that are important co-variates in PTSD. These particular assessments were then selected for their brevity, as the participants were already undergoing extensive testing in the scanner, and their reliability, as these are all well-established tests and frequently described in the literature. Within the PTSD group, there were significant rates of secondary co-morbid mental disorders, including major depressive disorder (MDD; 74% of the PTSD group). These findings are consistent with prevalence rates established through large scale studies in military populations [36]. Means and standard deviations for the groups are in Table 1.

### Procedure

Participants completed a version of the Intra-Extra Dimensional Set Shift (IED) Test [37] adapted for MEG [38]. Stimuli consisted of three coloured shapes, with two stimuli at the top of the screen and one stimulus at the bottom. There were 6 possible colours (yellow, cyan, green, red, blue, magenta) and 6 possible shapes (cross, circle, star, triangle, diamond, pentagon), which were combined to give 36 possible two-dimensional stimuli (e.g., cyan circle, cyan diamond, green circle, green star, etc.). The bottom image always matched one of the top images on one dimension, either shape or colour, but never both dimensions, and the match-target dimension was not indicated to the participant (see [34]).

For the task, using an MEG-compatible button-box, the participant pressed the left or right button to indicate whether the left or right top image matched the bottom image (2-alternative forced-choice). After 3–5 trials where the match parameter was the same (e.g., the colour ‘yellow’), the match parameter shifted. There were two types of shifts: intra-dimensional and extra-dimensional. Intra-dimensional shifts were within the same dimension, i.e., shift from colour-to-colour or shape-to-shape and easier, whereas extra-dimensional shifts were between dimensions, i.e., from colour-to-shape or shape-to-colour, and were more difficult, increasing attentional load and requiring greater cognitive control and mental flexibility.

370 trials requiring stimulus matching were presented, with a minimum of 3 trials presented before a shift rule was instantiated. Intra- and extra-dimensional shifts occurred in a pseudo-random order such that 50 shifts were intra-dimensional and 50 were extra-dimensional. Stimuli were presented using Presentation software (Neurobehavioral Systems, Inc., Berkeley CA) via a back projection screen placed 78 cm from the participants’ eyes. The stimuli were foveal and subtended 13° of arc (6.5° on either side of mid-line). The task was self-paced and each stimulus set was presented until a response was recorded, to a maximum of 4 s. The stimulus set was followed by a white fixation cross with a duration that was randomly jittered between 0.8–1.2 s. Responses were made on a response box with the thumbs of both hands, which would average out any unilateral hemispheric signal bias related to motor preparation and action. The entire test required a maximum of 30 minutes if 4 s was taken for each response; however, the average testing time was under 10 minutes.

### MEG data acquisition

MEG data were collected inside a magnetically-shielded room on a CTF Omega 151 channel system (CTF Systems, Inc., Coquitlam, Canada) at 600 Hz. Throughout the run, head position was continuously recorded by three fiducial coils placed on the nasion, and left and right pre-auricular points.

After the MEG session, anatomical 3T MRI images were acquired (Magnetom Tim Trio, Siemens AG, Erlangen, Germany), T1-weighted magnetic resonance images using high-resolution 3D MPRAGE sequences on a 12 channel head coil. MEG data were coregistered to the MRI structural images using the reference fiducial coil placements.

### MEG data processing

#### Seed definition and virtual electrode analyses

MEG data were band-pass filtered offline at 1–150 Hz, a notch filter applied at 60 Hz (8 Hz bandwidth) and a third-order spatial gradient environmental noise-cancellation applied to the recording. Ninety sources (seeds) were used, which constitute all cortical and subcortical sources in the Automated Anatomical Labeling (AAL) atlas [39], giving locations for time-series to be extracted and analyzed. Broadband time-series (‘virtual electrodes’) from these voxels were reconstructed using a vector beamformer on the basis of the 90 AAL coordinates for each subject and filtered into five classical EEG bandwidths for further analyses: Theta (4–7 Hz), Alpha (8–14 Hz), Beta (15–30 Hz), ‘low’ Gamma (30–80 Hz), and ‘high’ Gamma (80–150 Hz).

Beamformers are a type of spatial filter used to suppress signals from unwanted sources, whilst being optimally sensitive to activity in a given brain location (in this case, each of the 90 seed locations). Individual weight vectors are applied to each sensor measurement and summated to give an estimated source activity to a particular cortical seed location [40]. Additionally, MEG beamformers are effective at suppressing ocular artefacts generated by eye movements, and non-ocular artefacts, such as cardiac and muscle activity [41]; thus trial by trial rejection to eliminate eye movement and other such artefacts is not required.

#### Assessing functional connectivity: phase lag index

Each of the 5 band-pass filtered waveforms was then submitted to a functional connectivity analysis, using the phase lag index (PLI) [42]. The instantaneous phase of each sample from the filtered time-series was calculated using the Hilbert transform. The cross-trial degree of phase synchronisation for every time point between all pairwise combinations of the seeds was computed using the PLI, which is based on the magnitude of the imaginary component of the cross-spectrum [42]. Ranging between 0 and 1, these values quantify the phase synchrony between two sources, referred to as functional connectivity.

#### Statistical analysis

Adjacency matrices with PLI values acting as edge weights for all sources were constructed at every time point/sample, which resulted in a 90x90 [x5 frequencies x720 samples/time points] matrix of weighted undirected graphs for each participant. For the generation of statistically-thresholded functional connectivity images, temporally-averaged adjacency matrices over time windows of interest were generated, and statistical analyses were performed on the resulting matrices using the Network Based Statistic (NBS; [43]). NBS first applies an initial univariate threshold to each analyzed edge. The extent of connectivity components, defined as contiguous groups of nodes connected by suprathreshold connections, is then obtained. Group membership is then shuffled and the extent of the largest component which occurs in this surrogated data is then recorded, and this process is repeated 5000 times to generate a null distribution. The ranking of connectivity components from the unshuffled data in the surrogate distribution is used to determine statistical confidence; as the surrogate distribution considers the largest connectivity component that could occur, assuming the null hypothesis across the entire analyzed network, this approach is very effective in controlling for false positives due to multiple comparisons at any threshold. In the present analysis, the initial univariate threshold was set at a moderate t-value of 2.8, p < 0.004 (see [43,44]). Network measures of node strength were used to assess the importance of a node within the networks and these measures were obtained using Brain Connectivity Toolbox [45]. Brain networks were visualized using BrainNet Viewer [46]. Further analyses, such as correlations between psychometrics and graph properties, were completed using in-house scripts.
